# Imprinting and Editing of the Human CD4 T Cell Response to Influenza Virus

**DOI:** 10.3389/fimmu.2019.00932

**Published:** 2019-05-07

**Authors:** Sean A. Nelson, Andrea J. Sant

**Affiliations:** Department of Microbiology and Immunology, David H. Smith Center for Vaccine Biology and Immunology, University of Rochester Medical Center, Rochester, NY, United States

**Keywords:** CD4 T cells, vaccine, human immunology, Influenza virus, imprinting

## Abstract

Immunity to influenza is unique among pathogens, in that immune memory is established both via intermittent lung localized infections with highly variable influenza virus strains and by intramuscular vaccinations with inactivated protein-based vaccines. Studies in the past decades have suggested that the B cell responses to influenza infection and vaccination are highly biased by an individual's early history of influenza infection. This reactivity likely reflects both the competitive advantage that memory B cells have in an immune response and the relatively limited diversity of epitopes in influenza hemagglutinin that are recognized by B cells. In contrast, CD4 T cells recognize a wide array of epitopes, with specificities that are heavily influenced by the diversity of influenza antigens available, and a multiplicity of functions that are determined by both priming events and subsequent confrontations with antigens. Here, we consider the events that prime and remodel the influenza-specific CD4 T cell response in humans that have highly diverse immune histories and how the CD4 repertoire may be edited in terms of functional potential and viral epitope specificity. We discuss the consequences that imprinting and remodeling may have on the potential of different human hosts to rapidly respond with protective cellular immunity to infection. Finally, these issues are discussed in the context of future avenues of investigation and vaccine strategies.

## Overview

Immunological memory to influenza is established by infection and vaccination. Epidemiological studies suggest that children in North America are typically infected with seasonal influenza at a rate of 5–15% each year, depending on age and history of vaccination (1–3). In the U.S., it is now recommended that all children at 6 months of age and older receive yearly vaccination (4). Currently licensed vaccines include either intranasal inoculation of cold adapted influenza vaccines (CAIV), such as Flumist®, or inactivated influenza vaccine (IIV), delivered via intramuscular injection. Typically, the first vaccinations are with IIV, delivered in infants as sequential vaccinations separated by 28 days between prime and boost. After 2 years of age, children can be administered CAIV intranasally, with the goal of boosting cellular and local immunity in the respiratory tract. Thus, by many different mechanisms, CD4 T cells specific for influenza viral antigens are established early in life. Worldwide, most adults have likely first encountered influenza by infection, because influenza vaccines were not widely used until the last two decades. In contrast, most young children in the US could have been exposed to influenza antigens first by vaccination.

The human host confronts influenza antigens in diverse forms and at somewhat unpredictable intervals through periodic infections and yearly vaccinations. How these different types of encounters with influenza virus and its antigens affect CD4 T cell memory and phenotype is critically important to understand, because this accumulated memory will influence all subsequent responses. Despite the importance of this issue, currently our knowledge is quite limited. The concept of “imprinting” of influenza immunity has garnered a great deal of interest recently but this has largely been in the context of the B cell response (5–8). Here we consider the potential impact of CD4 T cell imprinting and editing of the human CD4 T cell repertoire to influenza and the potential consequences this might have on protective immunity to infection.

## Characteristics of the CD4 T Cell Response to Infection and Vaccination

Two aspects of the CD4 T cell response to infection are strikingly different from that of the B cell repertoire. First, the epitope specificity is tremendously diverse in human CD4 T cells, consisting of perhaps hundreds of different epitopes. This reactivity is determined in part by the multiple viral proteins targeted by CD4 T cells, stable binding of the antigenic peptide to MHC class II molecules (9–11) and by the precursor frequency of the CD4 T cells in the host to any given peptide (12). Even mice that express only one to two MHC class II molecules elicit CD4 T cells specific for 25–80 different peptide epitopes, distributed across both surface virion proteins such as hemagglutinin (HA) and neuramindase (NA), and internal virion proteins such as nucleoprotein (NP) and matrix protein (M1) (13–15). These antigen specificities have also been observed in humans (16–22). Due to expression of multiple HLA-class II isoforms and heterozygosity, humans can express as many as ten different class II molecules. As a result, they are likely to respond to a much wider array of peptide epitopes than do typical inbred mice. This complexity makes it extremely difficult to quantify reactivity to any particular influenza-derived peptide. Also complicating estimation of the diversity of the primary response of human CD4 T cells to infection are limitations in sampling tissues that are at the site of the response. Procedures are currently being developed to more broadly survey lymph nodes and the respiratory tract after infection (23–25). We believe that more efforts of this type are essential to understand the dynamic features of human immunity to influenza and long-term memory in the human host. However, at present, we can only estimate the breadth of human CD4 T cell immunity based on extrapolation of studies in animal models.

The second important distinction between human influenza-specific B cells and CD4 T cells is the functional complexity of the elicited response to infection. Accumulated studies to date have shown that the effector function and fate of CD4 T cells after priming by influenza infection are heterogeneous, and include follicular helper cells (“Tfh”), that remain in the lymph node for extended periods of time and facilitate B cell responses, prototypical Th1 cells that either enter recirculation or home to the lung to establish tissue resident memory, and cytotoxic CD4 T cells that are primarily detected in the respiratory tract [reviewed in (26)] (27, 28). Each of these subsets has distinct transcriptional profiles (29). The elements within the lung draining lymph node that control commitment to alternate fates of CD4 T cells are not well-understood. Differentiation decisions during CD4 T cell priming have been attributed to the local microenvironment, particularly cytokines (30, 31), but in the case of influenza infection, and dominant Th1 biased response, many other distinct functional subsets of CD4 T cells quickly emerge. Beyond the cytokine milieu, there are other parameters suggested to shape the CD4 T cell response to infection, including the impact of T cell receptor affinity (32, 33) and the epitope density that CD4 T cells encounter as they enter the antigen draining lymph node (34, 35).

In contrast to the diversity in specificity and functionality elicited by CD4 T cells in response to infection, vaccination with licensed vaccines is currently designed to elicit HA-specific neutralizing antibodies. Early vaccines were produced from isolated virions that were simply chemically inactivated prior to administration to humans (36). These early whole inactivated vaccines were highly immunogenic, likely due to the viral RNA content, and contained diverse influenza proteins (37). Since the 1960s, vaccine production has been progressively modified to be less reactogenic in order to increase compliance and safety, and to be more highly enriched for the HA protein, as our understanding of the role of neutralizing antibody in sterilizing protection from influenza has grown. Accordingly, the CD4 T cell responses to influenza vaccines have become focused in specificity and more limited in inflammatory response (38, 39). A recently licensed influenza vaccine now contains only pure HA proteins (Flublok® Quadrivalent) (40), with the relevant HA from each circulating strain isolated from transfected insect cells, thus further focusing the immune response to the HA proteins. Whether increasingly purified influenza vaccines endow the host with more or less protection from infection is not known at this time. This may ultimately limit the specificity of CD4 T cells to highly diverse HA proteins, diminishing cross protection against diverse influenza strains. Protein vaccines delivered in the absence of adjuvant to naïve individuals elicit CD4 T cells of limited functional complexity (41–44). Both of these features may limit the overall protective capacity induced by influenza vaccines.

## Imprinting and Editing in the CD4 T Cell Response Among Different Age Groups and Individuals

By imprinting, we refer to the possibility that certain types of influenza confrontations, determined by age (e.g., the very young) or type (e.g., infection), permanently bias subsequent responses. Editing refers to the possibility that the CD4 T cell repertoire is remodeled with each subsequent encounter with influenza viruses and vaccines. Knowledge of these issues is essential in order to both predict and potentially design new vaccines that most effectively poise the host for future immunity. Although imprinting in influenza immunity is most commonly discussed with regard to the B cell response, we propose here that imprinting may also have a dramatic impact on the specificity, phenotype and persistence of the CD4 T cell repertoire.

**Figure 1:**
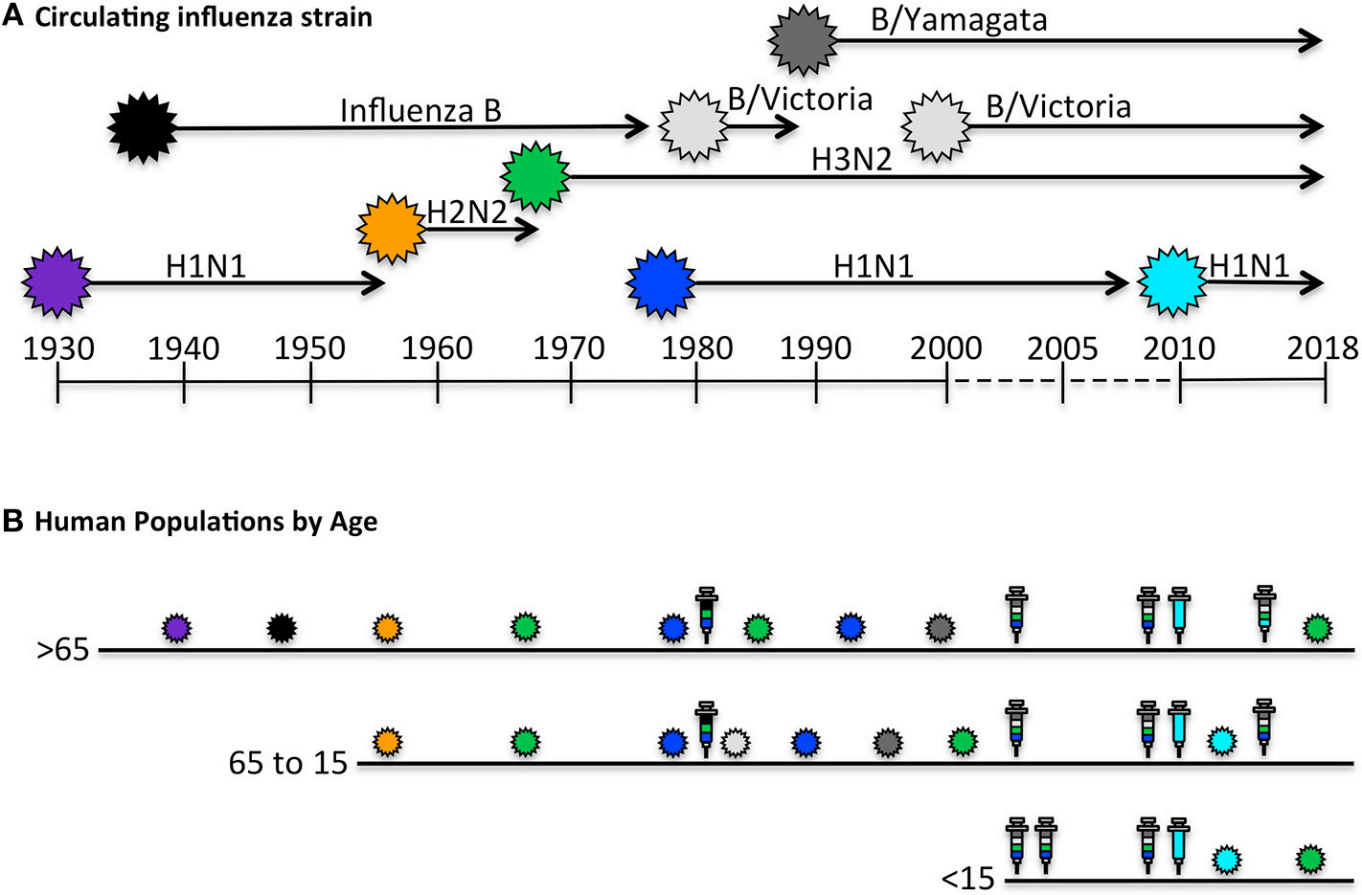
Human exposure to influenza viral antigens. **(A)** Shown are the seasonal influenza strains that have circulating since the first H1N1 virus was isolated in 1933 (45–47). At times, there has been only a single strain documented to be circulating, such as H1N1 from 1933 to 1957, after which H2N2 was circulating for approximately a decade. Influenza B was identified in approximately 1940 and has been co-circulating since, in different lineages (Victoria and Yamagata). Influenza H3N2 reappeared in 1968 and H1N1 began to recirculate with H3N2 in 1977. The H1N1 “seasonal” virus was replaced in 2009 with the novel pandemic “swine origin” virus which has dominated with H3N2 and influenza B in the last decade. **(B)** The human immune system encounters influenza antigens intermittently through both infection and vaccination, depicted by the colored influenza virions indicated in **(A)**, and in syringes, respectively. Seasonal influenza vaccines, shown in multiple colors, contain HA derived from each circulating strain, while pandemic vaccine formulations contain a single HA. Persons over 65 years of age, indicated in B, have had decades of exposure to distinct H1N1, H2N2, H3N2, and Influenza B isolates via infection, but limited exposure to vaccination until later in life, when we expect they would have already accumulated a diverse CD4 T cell repertoire. Persons 15–65 years of age have likely encountered diverse viral strains via infection, and depending on age, have likely had intermittent vaccinations. In contrast to older age group, the youngest age cohort (< 15 years old), may have had their first encounter with influenza derived antigens, especially HA, in the form of a prime-boost immunization. Thus, we predict that older adults would have a CD4 T cell repertoire with diverse antigen specificity and functional potential that was largely generated by infection, while younger individuals may have CD4 repertoire that is enriched in HA-specific cells and generated largely by vaccination and perhaps boosted periodically by mild infections. The specificity and function of the circulating memory populations in adults will depend on the factors discussed in the text.

Unlike animal models of infection or vaccination that might experience primary and perhaps secondary immune responses, the human immune system is primed and boosted with influenza antigens numerous times over a lifetime. Figure 1 illustrates the way these events may vary by the single parameter of age. The oldest individuals (>65 years of age) were likely exposed to influenza first through infection, and have had numerous subsequent exposures to distinct circulating H1N1, H2N2, H3N2, and influenza B viruses through infection [reviewed in (36, 47)]. Thus, based on periodic infections with different influenza viruses, we would expect that this oldest cohort of individuals would have accumulated a highly diverse CD4 T cell repertoire to distinct virus proteins. However, based on evolving vaccine recommendations, the immune repertoire of the over 65 cohort would have been perturbed by yearly vaccination for the past 1–2 decades [reviewed in (36, 48)]. Individuals in the 50–60 year old demographic may display the same pattern of early-life influenza virus exposure, but may not have received the yearly influenza vaccination suggested for older people. Conversely, children 15 years old and younger may have had their first confrontation with influenza through intramuscular vaccination with vaccines comprised of proteins from multiple virus strains, and enriched for HA. Whether and how frequently young children experience influenza infections is quite difficult to know with certainty, because many infections cause only mild disease, particularly among vaccinated individuals.

The simplest prediction of these scenarios is that older adults would have the largest epitope diversity of CD4 T cells, specific for many influenza virus proteins, with the most diverse functional potential, generated by each infection, while the youngest cohort might have a highly enriched HA-specific CD4 T cell repertoire generated largely by vaccination and perhaps boosted periodically by mild infections.

This simple model discussed above fails to account for several features of influenza immunity. First, in terms of the circulating repertoire of memory CD4 T cells that accumulates in humans, the potential requirement for periodic boosting to sustain CD4 T cell specificities is not clear. Also, it is not known if different functional subsets (e.g., Tfh vs. cytolytic cells) differ in this regard. Our own studies have shown that humans vaccinated with an H5N1 vaccine maintain some of the CD4 T cells specific for the unique H5-HA peptides for at least 5 years and that they can be recalled (49). This argues that if attrition does occur in humans, due to failure to boost, it is not complete within this time frame. Also, the impact of competition among CD4 T cell responses that likely occurs during complex immune responses, such as that induced by infection and vaccination is not yet well-understood, particularly during sequential, periodic confrontations (50, 51).

If intermittent boosting is required, some epitope specificities may become enriched for over time while others may decay. Current licensed inactivated vaccines typically contain some NP and M1, derived from the vaccine donor strain (52), which may be of sufficient quantity to boost pre-existing immunity generated by infection. Consequently, many humans may accumulate CD4 T cells specific for the most highly conserved epitopes within these internal virion proteins. The broad reactivity of these CD4 T cells could allow them to provide cross-reactive immunity against many influenza strains, particularly if their functional and lung homing potential induced by the original infection is maintained. Enrichment of these specificities over time with vaccination could be beneficial for the human host. If re-stimulation is required, then it is possible that unique epitopes in HA and NA proteins from viruses that are no longer in circulation disappear over time. Thus, the repertoire might be edited by “pruning” of some epitope specificities.

In support of the idea that adults may accumulate CD4 T cells specific for highly conserved HA-derived epitopes with age is a study showing that relative to younger subject, older adults display higher levels of highly conserved H1-reactive CD4 T cells, localized to epitopes mainly in the HA2 domain (53). In addition to the positive and negative effects of intermittent boosting of the CD4 T cells by conserved epitopes and losses due to attrition by neglect, it is also possible that there is loss of some potential epitope specificities due to the competitive advantage that memory cells have. Our laboratory has found that in sequential heterosubtypic infections in mice, CD4 T cells specific for NP epitopes that are conserved between the two viruses expand preferentially over new HA-derived epitope specificities present in the second virus (54), likely due to their higher abundance and greater sensitivity to antigen, both enhanced in memory T cells. Thus, editing of the CD4 T cell repertoire can depend on the sequence of viruses encountered. Also important to consider is that because of error prone polymerase in influenza virus, T cell epitopes in influenza proteins can accrue small mutations, leading to emergence of variants that may stimulate only a subset of the memory CD4 T cells. Documented evidence for this is more common with CD8 T cells because of the greater availability of MHC-peptide tetramers and well-defined short peptides of 8–10 amino acids, allowing easily deduced binding registers to MHC class I proteins. MHC class II molecules, in contrast, bind peptides of highly variable length (12–25 amino acids), due to a peptide binding pocket that is open at both ends and often have poorly delineated MHC binding registers. In animal models, well-defined variant peptides for CD4 T cells behave as altered peptide ligands, inducing modified functionality (55–59) or modified T cell receptor usage (60). An additional potential mechanism responsible for CD4 T cell repertoire editing, particularly after infection, are the possible negative effects of robust IFN-γ production on priming and expansion of new CD4 T cells. Human influenza-specific CD4 T cells in adults produce abundant IFN-γ (17, 18, 53, 61, 62) perhaps reflecting their original priming by infection. If these cells are recruited into the response to vaccination, elicitation of new CD4 T cell epitope specificities could be dampened via a complex network of suppression initiated by IFN-γ (10, 63, 64). It is known that T cell primed by infection can establish long-term memory in the respiratory tract (27, 65, 66), which endows them with the capacity for rapid protective responses to infection. It is possible that infection also seeds T cells in the periphery that preferentially return to the lung upon a secondary infection, based on their dominant Th1 phenotype and associated chemokine receptors (31) or priming via a lung draining antigen presenting cells after infection (67). Such infection-primed CD4 T cells may have priority for persistence as they were generated in the context of a robust inflammatory response and activation of many cells in the innate compartment.

## Essential Studies to Resolve the Impact of CD4 T Cell Imprinting and Editing in the Influenza Specific CD4 T Cell Repertoire

Resolution of the mechanisms that might underlie imprinting and editing of the CD4 T cell response is exciting to consider. First, and probably most informative, are longitudinal cohort studies that track the evolving immune response to infection and vaccination from early childhood to adulthood, where immune confrontations could be precisely monitored and documented (68). The best design would encompass both unvaccinated subjects, who will likely be primed first by infection and perhaps sequentially with different virus strains, and vaccinated subjects, who may have their first encounter with inactivated vaccines. Also critical in identifying factors that control imprinting will be improvements in approaches that allow low abundance human CD4 T cells, specific for single or selected epitopes from vaccines or viruses, to be quantified and characterized in these longitudinal studies. With refinement of these approaches, the functional fate and persistence of elicited CD4 T cells can be evaluated. For example, use of selected HLA-peptide tetramers coupled with either single cell sequencing or multiparameter flow cytometry would be extremely valuable. Finally, because of the potential of heterologous immunity—immunity generated by unrelated pathogens—to play a role in T cell responses (69, 70), it would be valuable to begin to develop methods to identify the array of pathogens and vaccines that an individual has been exposed to that may have shaped their existing T cell repertoire, an option that is feasible with carefully monitored cohorts. If immunological imprinting is unique to early childhood infection, then it is possible that some vaccine-specific responses in adults are drawn from heterologous infections established in childhood and then boosted by vaccination. This CD4 T cell repertoire may be distinct in several ways. First, the responses to vaccination might contribute to protection or lung pathology, depending on the effector phenotype elicited by the first infection (71, 72). Second, the cross- reactive response may have a narrowed breadth in TcR sequence, which might possess more limited efficacy and later cross-reactivity to variant influenza strains (70). With the help of advances in computational studies and data science, it may be possible to identify predictable events confronting the immune system that perturb and ultimately control the repertoire of CD4 T cells specific for influenza.

## The Possibility of Eliminating the “One Size Fits All” Vaccine Strategy

Currently, licensed vaccines are largely designed via a single platform with a limited and focused goal. Inactivated vaccines introduce HA from each circulating virus strain via intramuscular injection with the goal of eliciting neutralizing antibodies to the circulating influenza strains. The intranasal platform of Flumist, designed to provide more local and cellular immunity in the respiratory tract (73), has had uneven performance and appears to be most effective in young children (74). There have also been many recent initiatives to design vaccines that provide broadly protective immunity (75–80). With our increasing appreciation of the complexity and complementary nature of protective immunity to influenza, and specifically the multitude of functions that CD4 T cells play (27, 51, 81), there is likely to be increased focus on development of vaccines that prime or replenish particular specificities and functionalities. For example, if early-life exposures to influenza do effectively imprint the specificity and function of CD4 T cells, vaccines that establish the most robust and diverse repertoire of T cells may be most critical for infants and young children. In this regard, it is interesting to consider the potential consequence of widespread influenza vaccination beginning in infants. If childhood exposure is uniquely capable of imprinting specificity and functionality the immune system, then these early exposures to influenza primarily through vaccination might prime a limited CD4 repertoire. This repertoire could be enriched in HA reactivity Additionally, these CD4 T cells primed at peripheral sites without innate activators may have less lung homing potential and polyfunctionality and may instead be enriched for IL-2 or other Th2 biased responses, which are more typical of neonates (82). Conversely if adults who have received primarily inactivated, HA enriched vaccines are deficient in broadly reactive CD4 T cells, and are lacking established tissue resident memory cells, they may benefit from vaccine platforms that boost local immunity in the respiratory tract reactive with highly conserved proteins such as NP and M1. Alternate vaccine strategies for different individuals will require more sensitive and accurate approaches to define the components of the influenza specific immune repertoire that are deficient in the human host.

## Author Contributions

All authors listed have made a substantial, direct and intellectual contribution to the work, and approved it for publication.

### Conflict of Interest Statement

The authors declare that the research was conducted in the absence of any commercial or financial relationships that could be construed as a potential conflict of interest.
